# Modulation of Enzyme Activity in the Kynurenine Pathway by Kynurenine Monooxygenase Inhibition

**DOI:** 10.3389/fmolb.2019.00003

**Published:** 2019-02-08

**Authors:** Robert S. Phillips, Emma Carine Iradukunda, Tamera Hughes, J. Phillip Bowen

**Affiliations:** ^1^Department of Chemistry, University of Georgia, Athens, GA, United States; ^2^Department of Biochemistry, University of Georgia, Athens, GA, United States; ^3^Department of Pharmaceutical Sciences, College of Pharmacy, Mercer University, Atlanta, GA, United States

**Keywords:** kynurenine, kynurenine monooxygenase, kynurenine pathway, quinolinate, NMDA - receptor, inhibitor

## Abstract

The kynurenine pathway is the major route for tryptophan metabolism in mammals. Several of the metabolites in the kynurenine pathway, however, are potentially toxic, particularly 3-hydroxykynurenine, 3-hydroxyanthranilic acid, and quinolinic acid. Quinolinic acid (QUIN) is an excitotoxic agonist at the NMDA receptor, and has been shown to be elevated in neurodegenerative diseases such as Alzheimer's Disease and Huntington's Disease. Thus, inhibitors of enzymes in the kynurenine pathway may be valuable to treat these diseases. Kynurenine monooxygenase (KMO) is the ideal target for an inhibitor, since inhibition of it would be expected to decrease the toxic metabolites and increase kynurenic acid (KynA), which is neuroprotective. The first generation of KMO inhibitors was based on structural analogs of the substrate, L-kynurenine. These compounds showed reduction of QUIN and increased KynA *in vivo* in rats. After the determination of the x-ray crystal structure of yeast KMO, inhibitor design has been facilitated. Benzisoxazoles with sub-nM binding to KMO have been developed recently. Some KMO ligands promote the reaction of NADPH with O_2_ without hydroxylation, resulting in uncoupled formation of H_2_O_2_. This potentially toxic side reaction should be avoided in the design of drugs targeting the kynurenine pathway for treatment of neurodegenerative disorders.

## The Kynurenine Pathway

**Figure 1:**
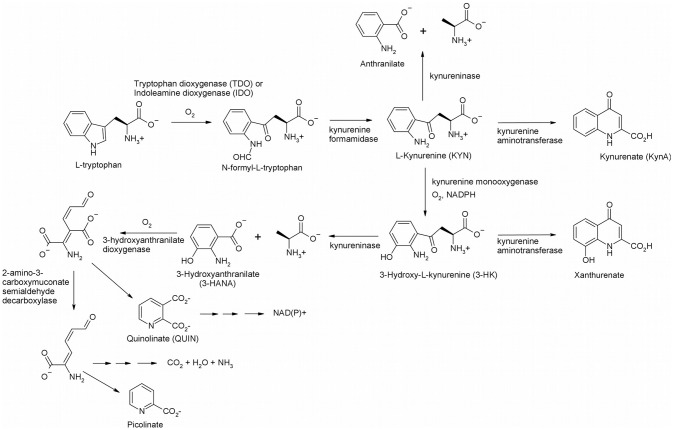
The kynurenine pathway in mammals.

The kynurenine pathway (KP) is the major pathway for tryptophan catabolism in mammals (Figure 1). In fact, as much as 95% of dietary tryptophan is catabolized via the KP (Botting, 1995). The KP has been implicated to play a major role in many diseases and disorders. These illnesses range from cancer to infectious diseases, such as HIV, neurological disorders such as schizophrenia (Erhardt et al., 2017) and depression (Réus et al., 2015); autoimmune diseases such as multiple sclerosis (Lovelace et al., 2016) and rheumatoid arthritis (Cribbs et al., 2014); peripheral conditions such as cardiovascular disease (Song et al., 2017) and acute pancreatitis (Mole et al., 2016); neurodegenerative diseases such as Huntington's disease (Sathyasaikumar et al., 2018), Alzheimer's disease (Giil et al., 2017), and Parkinson's disease (Lim et al., 2017).

The KP begins with oxygenation of L-tryptophan, catalyzed by one of two heme-containing dioxygenases, indoleamine 2, 3-dioxygenase (IDO) or tryptophan 2, 3-dioxygenase (TDO), to yield N-formyl-L-kynurenine. IDO exists ubiquitously in the body (Théate et al., 2015), and, while low quantities of TDO have been found in the brain, it is primarily expressed in the liver (Larkin et al., 2016). N-Formyl-L-kynurenine is rapidly converted by a formamidase to L-kynurenine (KYN), the eponymous intermediate of the KP. KYN can act as a substrate for three enzymes, kynurenine aminotransferase (KAT), kynureninase, and kynurenine 3-monooxygenase (KMO). When KYN undergoes metabolism via KAT it forms kynurenic acid (KynA), a known neuroprotective agent due to its binding to nicotinic acetylcholine receptors and antagonism on the NMDA, AMPA and kainite glutamate receptors (Perkins and Stone, 1982; Schwarcz et al., 2012; Vécsei et al., 2013). KYN can also react with kynureninase forming anthranilic acid, although it is a poor substrate (Lima et al., 2008). Kynurenine monooxygenase (KMO) converts KYN to 3-hydroxykynurenine (3-HK), which gives 3-hydroxyanthranilate (3-HANA) by the action of kynureninase. Another oxygenase, 3-hydroxyanthranilate dioxygenase, subsequently produces 2-amino-3-carboxymuconate semialdehyde, which undergoes spontaneous cyclization to quinolinate (QUIN) (Colabroy and Begley, 2005), ultimately leading to NAD(P)^+^ via quinolinate phosphoribosyltransferase. Decarboxylation of 2-amino-3-carboxymuconate semialdehyde initiates the pathway leading to complete catabolism and a branch leads to picolinate. It is important to note that there are several neuroactive intermediates in this pathway: KynA, QUIN, 3-HK, and 3-HANA. KynA is a known neuroprotective agent since it is an antagonist of the N-methyl D-aspartate (NMDA) receptor (Stone, 2000). The other neuroactive intermediates, QUIN, 3-HK, and 3-HANA, are known neurotoxic agents. 3-HK and 3-HANA serve as free-radical generators (Goldstein et al., 2000), while QUIN is an excitotoxic NMDA agonist (Stone and Perkins, 1981). Thus, modulation of enzyme activity in the KP will have effects on the NMDA receptor, which may be useful for treatment of neurodegenerative diseases that result from excessive

QUIN. The best drug target in the pathway is KMO, since blockage at this point will likely increase the neuroprotective KynA and decrease the neurotoxic metabolites, 3-HK, 3-HANA, and QUIN. The structure and inhibition of KMO has been reviewed recently (Dounay et al., 2015; Smith et al., 2016). In this review, we present the historical development of KMO inhibitors and review more recent developments.

In healthy tissue, the concentration of QUIN in the brain is low compared to blood and systemic tissues. An immune response, however, causes levels of QUIN to rise dramatically (Heyes et al., 1992). Macrophages, microglia and dendritic cells are the major generators of QUIN under inflammatory conditions. Astrocytes and neurons are capable of up taking and catabolizing QUIN. In this case, however, the catabolic system is easily saturated further resulting in the toxic accumulation of QUIN within the cells (Chen and Guillemin, 2009).

The toxicity caused by QUIN has been attributed to its ability to activate the neuronal NMDA subtype of glutamate receptors. While this remains true, additional mechanisms have also been shown to contribute to this complex neurotoxicity. QUIN is not only capable of potentiating it's own toxicity but also other excitotoxins such as glutamate, while inhibiting the reuptake of glutamate by astrocytes. QUIN compromises the integrity of the BBB, generates reactive oxygen intermediates, and depletes endogenous antioxidant and peroxidation of lipid molecules (Guillemin, 2012).

Increased production of nitric oxide has been shown in rodents and human neurons and astrocytes following induction of neuronal nitric oxide synthase by QUIN (Aguilera et al., 2007). Dysregulation of astroglial function and gliotoxicity is also proposed to augment QUIN's ability to kill neurons, redefining the cellular connection between neurons and glia in both physiological processes and pathological conditions (Lee et al., 2010). QUIN increases the phosphorylation of cellular structural proteins damaging the cytoskeleton of neurons and astrocytes (Pierozan et al., 2010). This destruction of cellular structure has brought significant interest to QUIN's role in hyperphosphorylated tau in Alzheimer's disease (AD) (Rahman et al., 2009).

Several studies have confirmed the pathological role QUIN plays in the development of many diseases. Elevated concentrations of QUIN have proven to directly contribute to HD, AD, AIDs related dementia (Chen and Guillemin, 2009), poliovirus brain infection (Allegri et al., 2012), multiple sclerosis (Aeinehband et al., 2016), cerebral ischemia (Saito et al., 2006), cerebral malaria (Dobbie et al., 2000), and epilepsy (Heyes et al., 1994).

## The Three Dimensional Structure of Kynurenine Monooxygenase (KMO)

KMO belongs to a family of NADPH dependent flavin monooxygenases (Okamoto et al., 1967). It is encoded by one gene, has an FAD coenzyme, utilizes either NADPH or NADH, releases NADP^+^/NAD^+^ after flavin reduction, and has one Rossmann fold dinucleotide binding domain, which categorizes it as a Class A flavoprotein aromatic hydroxylase (van Berkel et al., 2006; Crozier and Moran, 2007). Human KMO (hKMO) is 486 amino acids in length with a molecular weight approximately 50 kDa (Alberati-Giani et al., 1997; Breton et al., 2000). Eukaryotic sequences of KMO exhibit a C-terminal transmembrane helix about 50 residues long, which is responsible for binding to the outer membrane of mitochondria. As discussed above, due to its proposed involvement in a number of diseases, KMO has been purified and studied from several sources. Tissue distribution studies have shown that mammalian KMO is highly expressed in the liver and kidney, and in small amounts it has also been found in endothelial, macrophages, microglial, and monocytic cells. While expressed in a wide array of cell types, very low levels of KMO have been found in brain cells (Courtney and Scheel, 2010). The mammalian enzyme has been difficult to express and purify due to its membrane binding properties. Unfortunately, since it makes recombinant expression difficult, the C-terminal membrane anchor helix was found to be essential for activity of pig KMO (Hirai et al., 2010).

**Figure 2:**
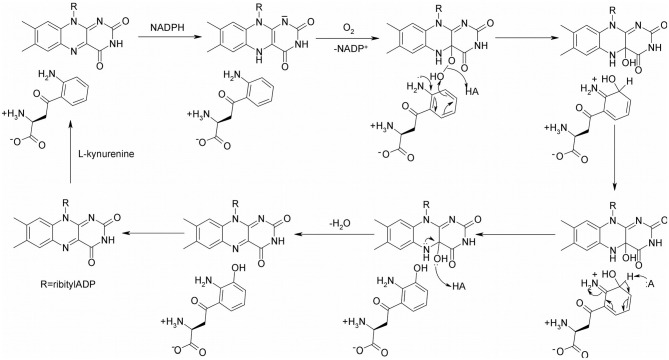
The proposed catalytic mechanism of KMO.

The proposed catalytic mechanism of KMO from *Pseudomonas fluorescens* is shown in Figure 2 (Crozier-Reabe et al., 2008). Like many oxidoreductases, the catalytic cycle of KMO can be divided into two half reactions, a reductive half and an oxidative half. The binding of KYN to KMO is relatively slow, making the reduction half of this reaction KYN dependent. Once kynurenine and NADPH bind to KMO, the FAD cofactor is reduced by NADPH, and NADP^+^ dissociates from the enzyme. The enzyme complex then reacts with molecular oxygen, forming a 4a-peroxyflavin intermediate that transfers an oxygen atom to the substrate. The resulting 4a-hydroxyflavin is rapidly dehydrated prior to product release. The oxidized enzyme complex subsequently undergoes a conformational change, facilitating the release of the product 3-HK, in the rate-limiting step of this mechanism. As a result of this conformational change, there is a change in the visible spectrum of the oxidized enzyme on product release.

The first crystal structure of KMO, published in *Nature* in 2013, was of the *Saccharomyces cerevisiae* enzyme (ScKMO) (PDB 4J36 and 4J33), truncated at the C-terminus (Amaral et al., 2013). The structure was determined not only in the free form, but also in complex with the tight-binding inhibitor, UPF648. Both structures were solved as a dimer with PDB 4J33 at a resolution of 1.82 Ȧ and PDB 4J36 at a resolution of 2.13Ȧ. The KMO structure, similar to other flavin-dependent hydroxylase structures, features a Rossmann fold domain for flavin adenine dinucleotide (FAD) binding that interacts with a part of the β-domain holding five β-sheets and four α-helices (Huijbers et al., 2014). It was found that UPF-648 binds closely to this domain, initiating a conformational change, precluding L-Kyn binding and therefore inhibiting KMO activity. Conserved residues, Arg83 and Tyr97, bind the UPF-648 carboxylate and conserved hydrophobic residues, Leu221, Leu234, Met230, Ile232, Phe246, Phe322, and Pro321, flank the aromatic dichlorobenzene moiety. Mutagenesis and functional assays have found these residues to be conserved across different organisms, allowing the translation of this work to hKMO. ScKMO and human KMO share 38% identity and 51% similarity. Thus, the structure of ScKMO has been a useful template for docking screens using virtual compound libraries and aiding in the development of novel inhibitor scaffolds.

**Figure 3:**
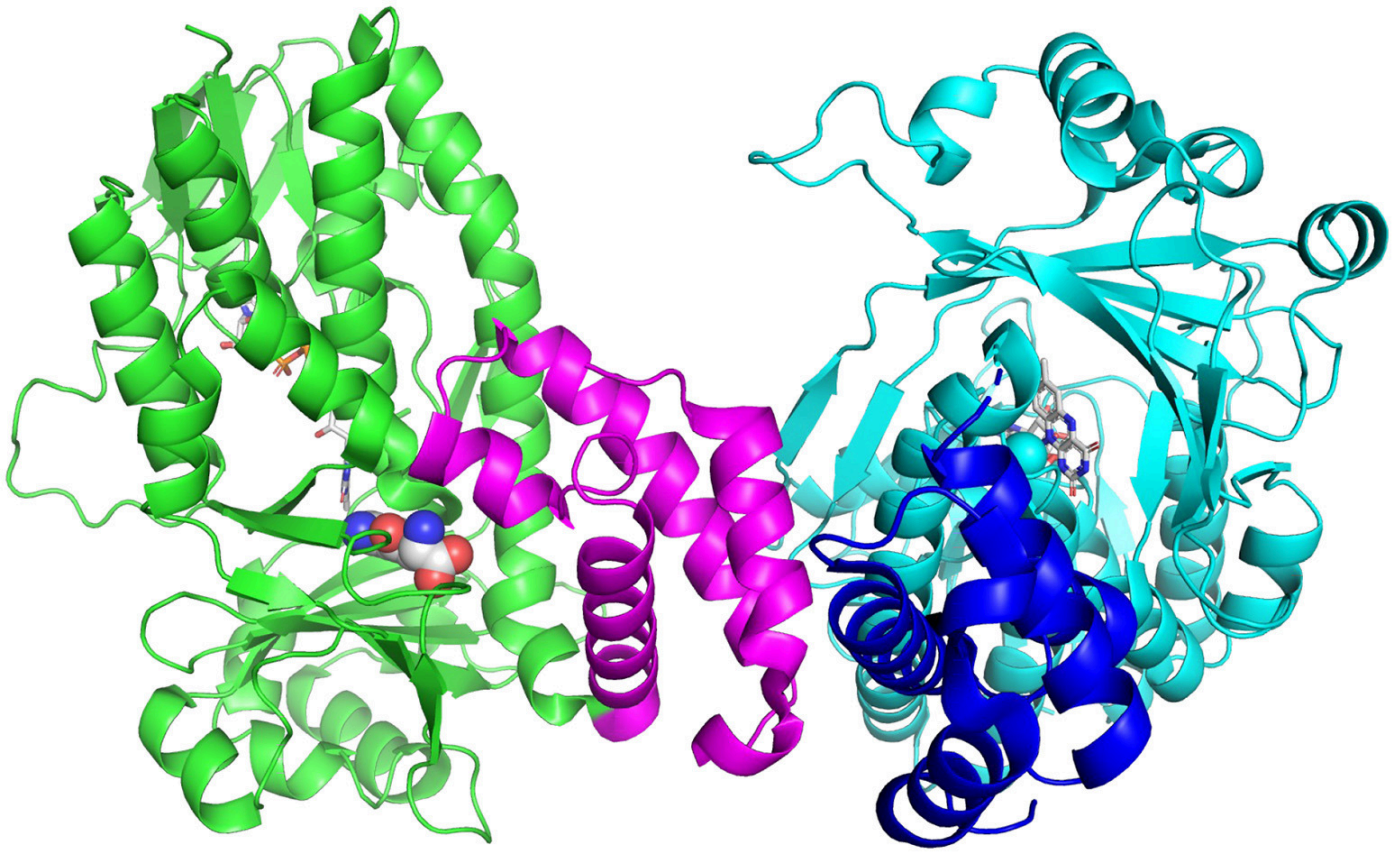
Structure of PfKMO with L-kynurenine bound (PDB 5NAK). L-Kynurenine (spheres) and FAD (sticks) are bound to the A-chain (green), while the B-chain (cyan) has only FAD bound (sticks). The C-terminal domain is shown in magenta (A chain) and blue (B-chain). This figure was prepared with Pymol (Version 2.1, Schrödinger, LLC).

Tryptophan catabolism via the KP has been identified in a number of bacteria, including *P. fluorescens, Cytophaga hutchinsonii* and *Ralstonia metallidurans* (Kurnasov et al., 2003). Soluble KMOs have been found in bacteria, *P. fluorescens* (Crozier and Moran, 2007) and *C. hutchisonii* (Kurnasov et al., 2003), which have facilitated mechanistic and structural studies. The enzyme from *P. fluorescens* (PfKMO) is a soluble enzyme with 37% identity to human KMO that can be expressed heterologously in *Escherichia coli* (Crozier and Moran, 2007). The crystal structures of PfKMO with a number of inhibitors and L-kynurenine bound have been solved recently (Hutchinson et al., 2017; Gao et al., 2018; Kim et al., 2018). The structure of PfKMO (Figure 3) is very similar to that of ScKMO. PfKMO contains two domains, with the main domain holding the Rossmann fold, the active site, the FAD cofactor and a C-terminal domain. Hydrophilic residues. Arg84, Tyr98, Tyr404, and Asn404, are close to the carboxylate groups of the substrate, and hydrophobic residues, Leu213, Leu226, Ile224, Phe238, and Met373, are close to the aromatic ring of the substrate. When L-kynurenine is in the active site, interactions between the carboxylate group and Arg84, Tyr98, Tyr404, and Asn369 are also present, revealing key interactions between PfKMO and substrates. These residues present in the active site of this enzyme are thought to be important in substrate binding and recognition. A significant conformational change was seen in the position of the C-terminal domain with substrate binding. For this reason, it was concluded that the C-terminal domain must play an integral role in the binding of substrates (Wilkinson, 2013; Gao et al., 2018). When PfKMO is not binding a substrate or inhibitor, the enzyme is said to be in an “open” conformation. It is theorized that this open conformation allows for accelerated binding of substrate and product release. Once a substrate binds to PfKMO, the C-terminal domain then moves to give a “closed” conformation, observed in the structure with L-kynurenine or inhibitors bound. Understanding the structural changes associated with substrate binding will be valuable in the development of effective KMO inhibitors.

The first structure of hKMO was reported recently (Kim et al., 2018). The crystal structure was solved to a resolution of 2.1 Å after engineering the deletion mutant, hKMO-374 (residues 1-374), in which the transmembrane domains were deleted, in order to obtain a human KMO protein suitable for crystallization. As described earlier (Hirai et al., 2010), hKMO, located in the mitochondrial outer membrane, contains two transmembrane domains (TMDs) and a C-terminal region responsible for mitochondrial targeting. As expected, the structure of hKMO is very similar to that of PfKMO and ScKMO. The first domain contains the FAD binding region, while the second contains the small N-terminal domain, consisting of alpha helices and an antiparallel beta sheet. However, hKMO-374 is inactive, in agreement with previous studies in which the transmembrane domains in pig and recombinant hKMO enzymes were required for enzymatic activity (Breton et al., 2000; Hirai et al., 2010). The findings from this study further provide insight into the KMO enzyme and will likely facilitate the development of KMO inhibitors.

## KMO Inhibitors

### Design of Inhibitors Before the KMO Crystal Structure

Two decades ago, when the first KMO inhibitors were evaluated, the crystal structure of KMO was still unknown, and therefore, inhibitor design was based on the structure of the KMO substrate, L-kynurenine (**1**), as a lead compound (Table 1). The desamino analog of L-kynurenine, β-benzoyl-L-alanine (**2**), was found to be a competitive inhibitor, with a *K*_d_ of 7.4 μM for PfKMO (Crozier-Reabe et al., 2008). Several of the first generation of KMO inhibitors have shown promising results in regulating NMDA receptor agonism and antagonism homeostasis. Among these compounds, (*m*-nitrobenzyl)alanine (*m*-NBA) (**3**) was the most potent inhibitor (IC_50_ = 0.9 μM) (Chiarugi et al., 1996). When 400 mg/kg of **3** was administered to rats, there was an increased level of both L-kynurenine and KYNA up to 10 times and 5 times, in the brain and blood, respectively. Inspired by those results, *m*-NBA was used as a lead compound to synthesize more potent inhibitors. (R,S)-3,4-Dichlorobenzoylalanine (FCE 28833A) (**4**), with an IC_50_ of 0.2 μM, was the most potent of a series of compounds prepared at Farmitalia Carlo Erba by adding substitutents to the benzene ring of **2**. **4** was found to have increased inhibition by almost 40-fold over **2** and 4.5-fold over **3**. *In vivo* studies with **4** were performed on rats with 400 mg/kg orally, and this showed an increase in both L-kynurenine and KYNA levels in brain tissue (Speciale et al., 1996). Ianthellamide A (**5**, IC_50_ = 1.5 μM) was isolated from the Australian sponge *Ianthella quadrangulata*. *In vivo* studies showed an increase of KYNA levels in rat brains following a systematic injection of 200 mg/kg (Feng et al., 2012). Kynurenines substituted at the 3-position were found to be competitive inhibitors with K_i_ values in the low μM region (Phillips et al., 2017). The most potent of these, 3,5-dibromo-L-kynurenine (**6**), is a competitive inhibitor with a K_i_ of 1.2 μM.

**Table 1:** Inhibitors of KMO.

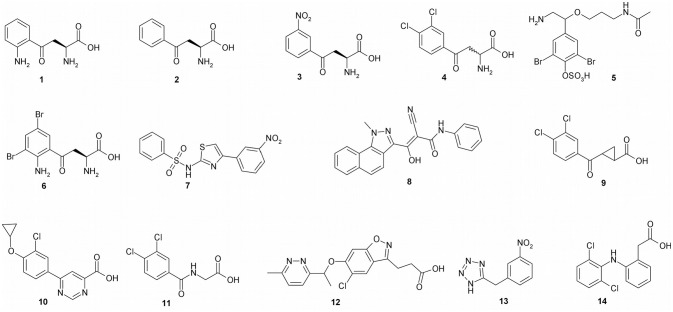				
**Compound**	**Name**	**IC**_**50**_ **or K**_**d**_ (**μM**)	**Tested for neurodegeneration**	**Peroxide production**
1	L-Kynurenine (substrate)	17.4	–	No
2	β-Benzoyl-L-alanine	7.6	–	Yes
3	*m*-NBA	0.90	Yes	Yes
4	FCE28833A	0.20	Yes	–
5	Ianthellamide A	1.5	–	–
6	3,5-Dibromo-L-kynurenine	1.2	–	–
7	Ro-61-8048	0.037	Yes	Yes
8	PNU-168754	0.040	–	–
9	UPF-648	0.020	Yes	–
10	CHDI-340246	0.0005	–	–
11	3,4-Dichlorohippuric acid	34	–	–
12	GSK 366	0.0007	–	–
13	5-(3-nitrobenzyl)-1H-tetrazole	6.3	–	–
14	Diclofenac	13.6	–	–

Structurally different sulfonamides were then examined and exhibited much stronger inhibitory potency compared to the previously examined scaffolds. Ro61-8048 (**7**) was the most active in this group, with inhibition in the nanomolar range (IC_50_ = 37 nM) (Röver et al., 1997). This compound has been shown to raise both L-kynurenine and KYNA levels in the brain through peripheral KMO inhibition, since it does not cross the blood brain barrier. JM6, a prodrug of Ro61-8048, was shown to reduce neurodegeneration in an Alzheimer's mouse model, despite not crossing the blood-brain barrier (Zwilling et al., 2011). This suggests that modulation of NMDA receptor activity can be achieved by compounds that do not cross the blood brain barrier. A series of tricyclic 3-oxo-propanenitriles compounds were patented, and one member of this series, PNU-168754 (**8**), has an IC50 of 40 nM (Pevarello et al., 1999). After determining that the α-amino group is not required for inhibition, and that the acid moiety is indeed essential for inhibition, a number of 4-aryl-4-oxobutanoic acids derivatives were prepared, with UPF-648 (**9**) being the best among them (IC_50_ = 20 nM). Treatment of mice with 100 μM UPF-648 has been shown to shift the KP to the synthesis of KYNA (Sapko et al., 2006). This compound was later used in the first KMO crystal structure to be solved (Amaral et al., 2013). This achievement marked the beginning of structure-based drug design of KMO.

### Design of Inhibitors After the KMO Crystal Structure

After the KMO structure determination, it became possible to design KMO inhibitors computationally with more precision. The first class of KMO inhibitors designed using the structural data was arylpyrimidine carboxylic acids. These can be considered as cyclic rigid analogs of kynurenine. The N3 is thought to mimic the L-kynurenine carbonyl oxygen, whereas the N1 mimics the amine group that is very essential for the inhibition. Compound **10** (IC_50_ = 0.5 nM) was found to be the best compound. It is highly selective for KMO over other enzymes in the KP, and has shown potency both *in vivo* and *in vitro*. When 10 mg/kg of **10** was administered orally in rats, an increase in L-kynurenine and KYNA, and a decrease of both QUIN and 3HK, in the brain was observed (Toledo-Sherman et al., 2015). Computational studies have been performed on arylpyrimidine carboxylic acids, predicting new derivatives with possible high inhibitory activity for KMO (Amin et al., 2016)

**Figure 4:**
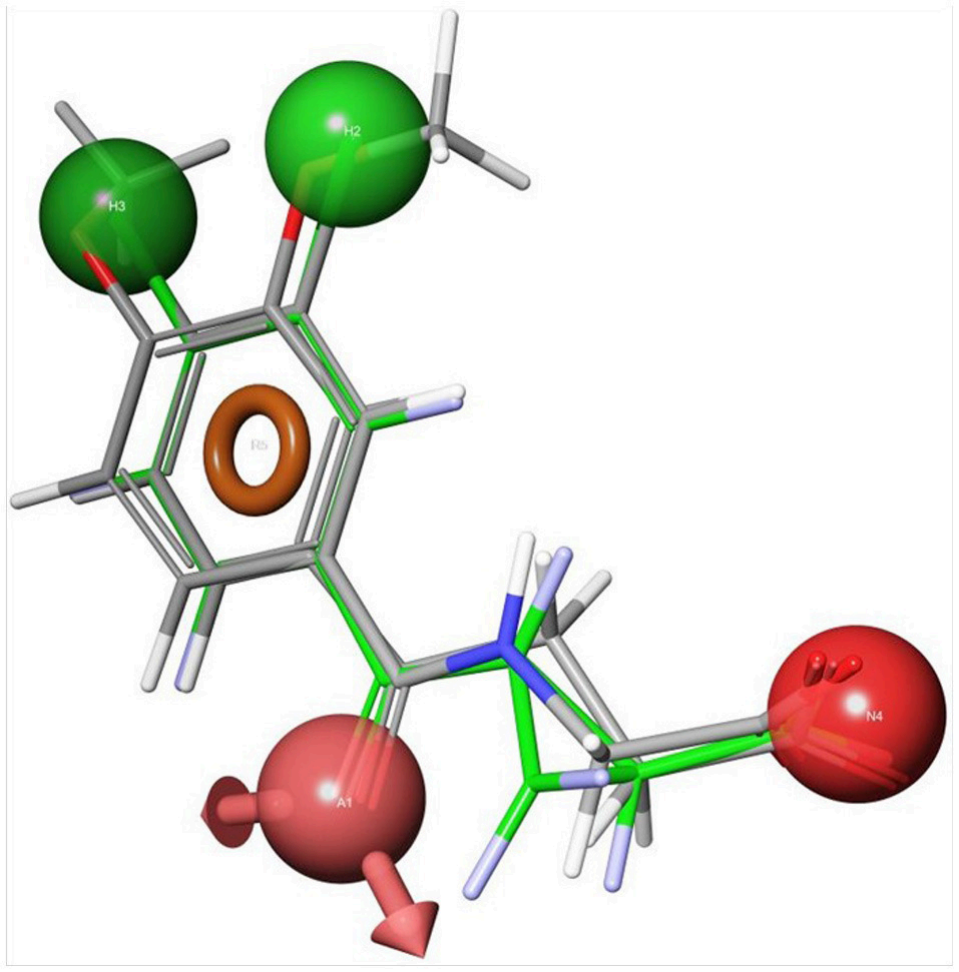
Receptor-based pharmacophoric models of 3,4-dimethoxyhippuric acid (**9**) aligned with the conformation of UPF648 as bound to ScKMO (pdb code: 4J36) (Phillips et al., 2017). The green spheres are hydrophobic centers, the red spheres are acidic centers, the pink spheres with arrows are H-bond acceptors, and the orange rings are aromatic planes.

Using the first ScKMO structure (Amaral et al., 2013) and an already known KMO inhibitor, UPF 648 (9), another set of compounds were proposed using a pharmacophore (Figure 4). This pharmacophore allowed prediction of a set of compounds that fit the active site of the KMO as **7** does. Among several predicted inhibitors, 3,4-dichlorohippuric acid (**11**) showed greatest inhibitory potency *in vitro* (K_i_ = 34 μM) (Phillips et al., 2017).

Benzisoxazoles are the most potent KMO inhibitors found so far. They were examined for inhibitory potency against KMO, with the goal of targeting acute pancreatitis and multiple organ dysfunction syndrome (Hutchinson et al., 2017). They are similar in structure to L-kynurenine and have inhibitory potency in the nanomolar range. GSK 366 (Table 1, **Compound 12)** is by far the most potent among them, with IC_50_ values of 2.3 nM and 0.7 nM for hKMO and PfKMO, respectively. This series of compounds contain chlorine, which was previously determined to play an important role in inhibiting KMO in **4**, **8**, and **10** (Breton et al., 2000; Hutchinson et al., 2017). This compound binds to the active site, and the methylpyridazine ring on the benzisoxazole tilts the flavin, which is thought to promote the potency and residence time of the inhibition. Though they have not been studied yet for their potency against neurodegeneration, notably the ability to cross the blood brain barrier, they still provide exciting insights and serve as great lead compounds for the future drug design and discovery for neurodegenerative disorders.

High throughput screening of a series of tetrazoles was performed with RapidFire mass spectrometry. The best compound identified was 5-(3-nitrobenzyl)-1H-tetrazole (**13**), with an IC_50_ of 6.3 μM *(*Lowe et al., 2014). Recently, another group proposed a molecular similarity and drug repurposing approach. Known drugs were computationally tested for KMO inhibitory capacities. Through this ligand-based approach, diclofenac (Table 1, **Compound 14**), a known anti-inflammatory drug, has been identified as a KMO inhibitor (IC_50_ = 13.6 μM) (Shave et al., 2018). This study sets the stage for future studies using molecular similarities studies as well as drug repurposing. The properties of various types of KMO inhibitors are summarized in Table 1.

### Limitations

Though structural analogs of L-kynurenine show inhibition, some of them have shown the potential of causing life threatening side effects, since they were found to generate cytotoxic hydrogen peroxide through futile cycles of flavin reduction and oxidation. Upon binding, NADPH reduces FAD and leaves as NADP^+^, then the oxygen molecule binds and forms an L-Kynurenine-FAD-hydroperoxide complex intermediate (Figure 2). The hydroxylation of L-kynurenine then proceeds to make water and 3-HK. Since these inhibitors have structural similarities to L-kynurenine, but are not capable of hydroxylation, some potential KMO inhibitors are uncouplers of NADPH oxidation (Crozier-Reabe et al., 2008). In the presence of *m*-NBA, and UPF-648, there is an accumulation of the hydroperoxyflavin, which decays to yield hydrogen peroxide. A recent study provided a better understanding on how the uncoupling happens (Kim et al., 2018). The authors proposed the flavin reduction in KMO is associated with conformational changes, via π- π interactions between a substrate or an NADPH uncoupler, and the loop above the *re-*side of the flavin. They determined that the substrate binding precedes flavin reduction. Ro61-8048 has shown interesting results where it acts as a simple competitive inhibitor in ScKMO and hKMO, and an NADPH uncoupler in PfKMO. This is is due to different binding modes this compound has in those two species. It binds in the active site of ScKMO but does not induce conformational changes in the protein the same way it does in PfKMO. Thus, it is important in the design of KMO inhibitors for treatment of neurodegenerative diseases to avoid compounds that will act as uncouplers of NADPH oxidation, generating highly toxic reactive oxygen species.

## Author Contributions

All authors listed have made a substantial, direct and intellectual contribution to the work, and approved it for publication.

### Conflict of Interest Statement

The authors declare that the research was conducted in the absence of any commercial or financial relationships that could be construed as a potential conflict of interest.
